# Reply to Singh et al

**DOI:** 10.1093/icvts/ivag110

**Published:** 2026-04-13

**Authors:** Sanae Kuroda, Wataru Nishio

**Affiliations:** Division of Chest Surgery, Hyogo Cancer Center, Akashi City 673-8558, Japan; Division of Chest Surgery, Hyogo Cancer Center, Akashi City 673-8558, Japan

We thank Singh and Srivastav for their thoughtful comments on our recent study examining postoperative pulmonary function and structural remodelling after lobectomy in patients with and without chronic obstructive pulmonary disease (COPD). Their observations provide valuable perspectives that help further interpretation of our findings.

First, the authors raise an important point regarding the interpretation of the higher measured-to-predicted FEV1.0 ratio (MPFR) observed in patients with COPD. As noted, predicted postoperative FEV1.0 was estimated using CT volumetry, and this approach may be influenced by heterogeneous emphysematous changes. In COPD lungs, volumetric prediction may underestimate the functional contribution of relatively preserved regions. In addition, resection of severely emphysematous and functionally impaired lung tissue may produce an effect analogous to lung volume reduction surgery, improving ventilatory efficiency. Therefore, the higher MPFR observed in the COPD group likely reflects a combination of methodological factors related to prediction models and physiological improvements following resection of diseased lung tissue.

Second, Singh and Srivastav highlight the dissociation between relatively stable D-values and the increase in low-attenuation area (LAA), particularly in patients without COPD. As discussed in our study, the D-value represents the complexity of alveolar structural distribution and is relatively stable once substantial emphysematous destruction has occurred. In contrast, LAA is more sensitive to volumetric and mechanical changes in the lung. The increase in LAA observed in the non-COPD group likely reflects compensatory expansion of the remaining lung after lobectomy rather than progression of emphysematous destruction. The stability of the D-value in both groups suggests that microstructural architectural complexity remains largely preserved during postoperative adaptation.

Third, regarding the subgroup analysis according to the emphysematous status of the resected lobe, the relatively consistent MPFR across groups suggests that postoperative functional preservation is influenced more strongly by overall lung mechanics than by the regional distribution of emphysema. This finding supports the concept that postoperative pulmonary function is determined by integrated whole-lung adaptation rather than by localized anatomical characteristics of the resected lobe.

Finally, we agree that additional physiological variables, such as lung volumes or indices related to dynamic hyperinflation, may further refine predictive models of postoperative function. Future investigations integrating structural imaging, physiological measurements, and longitudinal assessments may provide deeper insight into the mechanisms of postoperative lung remodelling.

We appreciate the authors’ interest in our work and their constructive comments, which contribute to further discussion on computed tomography (CT)-based assessment of postoperative lung adaptation.
